# Evolutionary transition towards permanent chloroplasts? - Division of kleptochloroplasts in starved cells of two species of *Dinophysis* (Dinophyceae)

**DOI:** 10.1371/journal.pone.0177512

**Published:** 2017-05-11

**Authors:** Pernille Møller Rusterholz, Per Juel Hansen, Niels Daugbjerg

**Affiliations:** Marine Biological Section, Department of Biology, University of Copenhagen, Copenhagen, Denmark; University of Connecticut, UNITED STATES

## Abstract

Species within the marine toxic dinoflagellate genus *Dinophysis* are phagotrophic organisms that exploit chloroplasts (kleptochloroplasts) from other protists to perform photosynthesis. *Dinophysis* spp. acquire the kleptochloroplasts from the ciliate *Mesodinium rubrum*, which in turn acquires the chloroplasts from a unique clade of cryptophytes. *Dinophysis* spp. digest the prey nuclei and all other cell organelles upon ingestion (except the kleptochloroplasts) and they are therefore believed to constantly acquire new chloroplasts as the populations grow. Previous studies have, however, indicated that *Dinophysis* can keep the kleptochloroplasts active during long term starvation and are able to produce photosynthetic pigments when exposed to prey starvation. This indicates a considerable control over the kleptochloroplasts and the ability of *Dinophysis* to replicate its kleptochloroplasts was therefore re-investigated in detail in this study. The kleptochloroplasts of *Dinophysis acuta* and *Dinophysis acuminata* were analyzed using confocal microscopy and 3D bioimaging software during long term starvation experiments. The cell concentrations were monitored to confirm cell divisions and samples were withdrawn each time a doubling had occurred. The results show direct evidence of kleptochloroplastidic division and that the decreases in total kleptochloroplast volume, number of kleptochloroplasts and number of kleptochloroplast centers were not caused by dilution due to cell divisions. This is the first report of division of kleptochloroplasts in any protist without the associated prey nuclei. This indicates that *Dinophysis* spp. may be in a transitional phase towards possessing permanent chloroplasts, which thereby potentially makes it a key organism to understand the evolution of phototrophic protists.

## Introduction

Acquisition of phototrophy in the form of endosymbiosis dates back to the first origin of all eukaryotic lineages. The lineages have further evolved by a series of endosymbioses and probably also other types of organelle retention. Yet, we understand only parts of the genetic events that led to the phototrophic organisms we know today [1]. The organisms that do not possess permanent chloroplasts, but still are capable of performing photosynthesis by the exploitation of other phototrophic organisms, may contain some of the answers to the gaps in the plastid evolution by revealing transitional phases, and potential pathways of horizontal gene transfer.

Phototrophic exploitation by phagotrophic organisms is commonly found in both marine and freshwater and especially seen in the so-called SAR lineage comprising Stramenopiles, Alveolates and Rhizaria (e.g. ciliates, dinoflagellates, radiolarians and foraminiferans) [2]. Such exploitation can, besides endosymbiosis, be displayed in two ways. Most commonly the prey nucleus (kleptokaryon) is retained along with the chloroplasts allowing the host to gain regulatory abilities, while the genetic machinery to maintain the chloroplasts are kept. Other hosts only sequester the kleptochloroplasts while the rest of the prey is quickly digested upon ingestion. Here, the association between the prey-chloroplast and the host is often temporary due to the lack of regulatory organelles and the kleptochloroplasts are only retained for a short time, i.e. hours to days [2].

Two examples of kleptochloroplastidic dinoflagellates are studied here; *Dinophysis acuta* and *Dinophysis acuminata*. *Dinophysis* spp. retain the kleptochloroplasts from their prey, the red pigmented *Mesodinium* spp., which in turn receive them from ingested cryptophytes within the *Teleaulax/Geminigera* clade. *Dinophysis* ingests the ciliates’ cytoplasm, chloroplasts and other organelles by means of a feeding tube (a peduncle) that penetrates the ciliate prey. The content is then transferred to a food vacuole within the dinoflagellate and all of it is digested except for the kleptochloroplasts [3]. Research in chloroplast regulating genes within *Dinophysis* spp. is very limited as only one study so far has documented plastid encoded genes within the genome [4]. Five such genes were expressed in *D*. *acuminata* and only one of these originated from cryptophytes [4]. The low amount of plastid encoded genes in *Dinophysis* spp. indicates a limited control over the kleptochloroplasts. Also, cell cycle analyses in *D*. *norvegica* have indicated that *Dinophysis* spp. cannot divide the sequestered kleptochloroplasts [5,6]. Thus, these data suggest that *Dinophysis* spp. have limited control of its retained chloroplasts.

Recent studies however have demonstrated that *Dinophysis* spp. have considerable morphological and physiological control of its kleptochloroplasts. Following ingestion of the cellular contents of *M*. *rubrum* the kleptochloroplasts are redistributed to kleptochloroplast centers in the periphery of the cell [3]. Upon this incorporation into the dinoflagellate the kleptochloroplasts undergo several structural changes, for example positioning the pyrenoids terminally to form these kleptochloroplast centers [7]. Physiological studies have indicated that *Dinophysis* spp. are able to keep the kleptochloroplasts functionally active in cells that are starved of their prey for an extended time period [8–11]. Recently it was also documented that *Dinophysis acuta* can photoregulate, i.e. cells can maintain levels of chlorophyll *a* and phycoerythrin as well as photoprotective pigments during several cell divisions in prey starved cultures [12]. Photoacclimation such as expressed in truly phototrophic algae was not documented, as no changes were observed in photosynthesis vs. irradiance parameters, e.g. maximum electron transport (mETR) or in the slope of ETR versus irradiance (αETR). Overall, however, these recent studies indicate considerable morphological and physiological control of the kleptochloroplasts.

The findings that *Dinophysis* spp. can produce photosynthetic and photoprotective pigments and maintain cellular levels of these pigments in prey starved cultures at low irradiance after several cell divisions indicate control of the kleptochloroplasts and suggest the possible ability of *Dinophysis* spp. to replicate the kleptochloroplasts. This is in contrast to current beliefs. Hence, if *Dinophysis* is able to replicate its kleptochloroplasts this would be the first time ever this has been observed among protists that only sequester the chloroplasts (and not nuclear material).

The present study aimed to document the fate of the cryptophyte kleptochloroplasts in two common species of *Dinophysis*, *D*. *acuta* and *D*. *acuminata* using confocal microscopy followed by 3D bioimaging analyses at various irradiances. The two species were incubated at different irradiances during prey starvation to study the effect of irradiance on the 1) number of kleptochloroplasts, 2) number of kleptochloroplast centers and 3) total kleptochloroplast volume. We hypothesized that (H1) the two *Dinophysis* species can divide the kleptochloroplasts, and the kleptochloroplast centers. We further hypothesized (H2) that the number of chloroplasts, chloroplast centers and the total kleptochloroplast volume generally would decrease during long-term starvation, but that this is not directly linked to cell divisions and (H3) that the reduction in the number of kleptochloroplasts, kleptochloroplast centers and the total kleptochloroplast volume are more pronounced in cells grown at high irradiances compared to those grown at low irradiances.

## Materials and methods

### Cultures and their origin

Four different cultures (*Dinophysis acuta*, *D*. *acuminata*, *Mesodinium rubrum* and *Teleaulax amphioxeia*) were grown in f/2 medium (N source is nitrate) with a salinity of 30 and a pH 8.2 ± 1 [13] and kept in temperature regulated climate rooms at 15°C ± 1.0 with a light:dark cycle of 16:8 h. The cultures of *Dinophysis* were fed twice a week with *M*. *rubrum* in a predator:prey ratio of 1:5–10. The culture of *M*. *rubrum* was regularly fed with *T*. *amphioxeia* at a predator:prey ratio of 1:5. Every 1–2 weeks the cultures were inoculated into fresh f/2 medium.

The culture of *D*. *acuminata* was established from a surface water sample (Hvalpsund, Denmark), in June 2007 with a temperature of 18°C and a salinity of 22. Cells were isolated with a hand drawn glass pipette, and at least 100 cells were picked and rinsed several times in filtered seawater (0.20 μm). Successful cultures were kept in multi-well dishes and provided with *M*. *rubrum* as prey [9]. The culture of *D*. *acuta* was established from a water sample collected in the North Atlantic in June 2010. Cells were picked using a hand drawn glass pipette and washed in filtered seawater. The successful cultures were kept and provided with *M*. *rubrum* as prey [11]. The cultures of the ciliate *M*. *rubrum* (MBL-DK2009) and cryptophyte *T*. *amphioxeia* (SCCAP K-1837) were isolated from water samples collected in Helsingør Harbor, Denmark, in September 2009.

### Illumination

The illumination was provided by cool white fluorescent light tubes fixed above or below the cultures (see later). The photon flux for the different light intensity treatments were measured in air with a spherical PAR sensor (Walz, ULM-500).

### pH measurements

pH was measured directly in the Nunc flasks every second day during the experiments using a pH meter (pHenomenal pH 1100L, VWR). Once a week the pH-meter was calibrated in 3 standard buffers (pH of 4.01, 7.0 and 10.0). When pH in the starved cultures approached 8.5 an inverse filtration was performed by removing 80% of the media through a 10 μm filter, and replacing it with fresh media. This procedure was repeated at least 3 times and following this, cell concentrations were enumerated and adjusted (if needed) to the same level as before filtration.

### Cell enumeration

Cell concentrations were enumerated throughout the experiment after fixation in Lugol’s solution (1% final concentration). A minimum of 200 cells were counted using Sedgewick Rafter chambers.

### Experimental design

**Fig 1:**
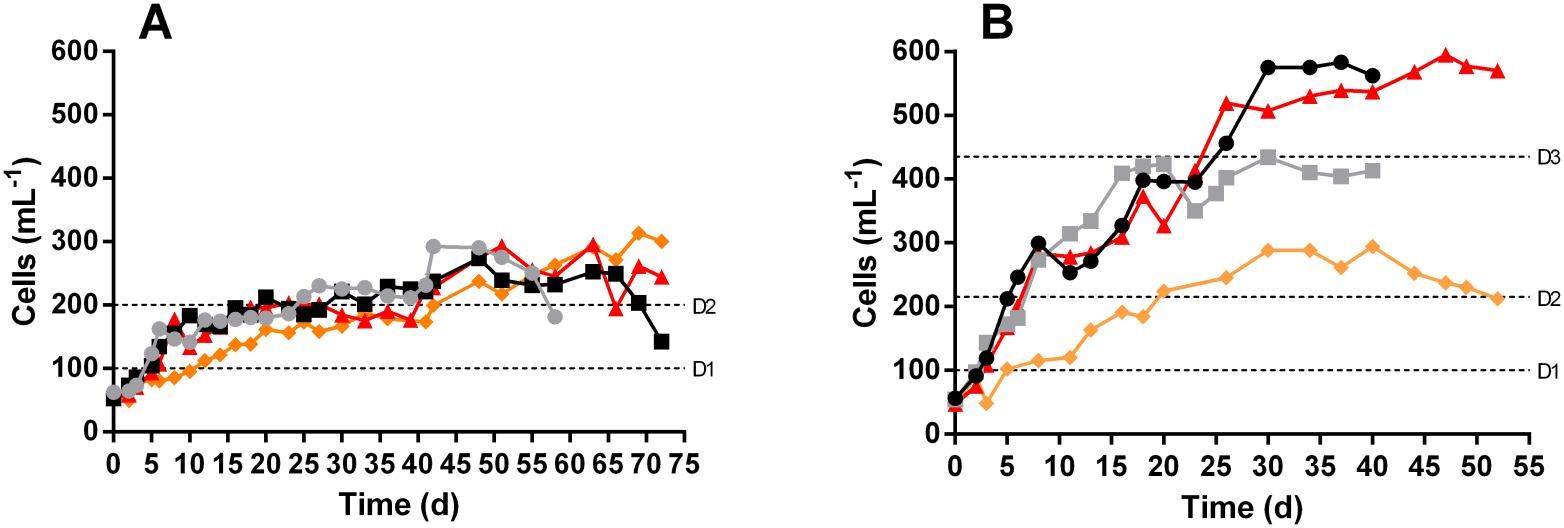
Development of cell concentrations of *D*. *acuta* (A) and *D*. *acuminata* (B) during prey starvation at four different light incubations. The light incubations are 100_i_ (black circles), 75_i_ (grey squares), 50_i_ (red triangles), 25_i_ (orange diamonds). The dashed lines indicate the approximate doublings in the concentrations for the first (D1), second (D2) and third (D3) division.

Prey saturated (i.e. predator:prey ratio of 1:5–10) cultures of *D*. *acuminata* and *D*. *acuta* grown under 100 μmol photons m^-2^ s^-1^ were allowed to deplete their prey and then subdivided into 4 different light incubations: 100, 75, 50, and 25 μmol photons m^-2^ s^-1^, hereafter referred to as 100_i_, 75_i_, 50_i_ and 25_i_. For 25_i_ incubations the light was provided from above whereas for the 50_i_, 75_i_ and 100_i_ incubations the light was primarily provided from below. The incubations were starved from day 0 to ensure that new chloroplast material was not obtained by the cells during the experiment. Cell concentrations (Fig 1) and pH (S1 Fig) were monitored every second day to ensure that samplings would be taken right after a doubling had occurred. Samples were also collected on day 0 and at the end of the experimental period.

### Sampling and photodocumentation of kleptochloroplasts

For each sampling, cells were fixed in 1% glutaraldehyde. The fixed samples were then filtered onto 0.2 μm black polycarbonate filters and afterwards mounted on glass slides with immersion oil (Olympus, Japan). Analysis and photography of minimum 20 randomly picked cells per sample were conducted in an inverted microscope (Olympus IX81, Japan) equipped with a spinning-disc unit for confocal imaging. The autofluorescence of the kleptochloroplasts in *D*. *acuminata* and *D*. *acuta* was visualized by excitation with green light (530–550 nm) using the CellR software (Olympus, Japan). Photographs were taken with a b/w digital FViewII camera (Olympus Soft Imaging System, Tokyo, Japan) and fluorescent data of the cells were taken as z-stacks with a distance of 0.2 μm between the individual focal planes. For reconstruction and measurements of the three-dimensional models, the software Imaris 8.2 (Bitplane AG, Zürich, Switzerland) was used.

### Statistics

One-way and two-way ANOVA followed by Tukey’s multi comparison tests were performed in Graphpad Prism (ver. 6.0). For all the statistical tests an alpha-limit of 0.05 was used.

## Results

### Population dynamics

During the experimental period, cell concentrations at all irradiances increased from the initial 50 to 270–310 cells mL^-1^ for *D*. *acuta* and up to 294–595 cells mL^-1^ for *D*. *acuminata*. A doubling in cell abundance thus occurred twice in all incubations (Fig 1A and 1B). A third doubling was observed only in two incubations (100_i_ and 50_i_) of *D*. *acuminata*. The incubations were monitored until a decrease in cell concentration was observed, or when more than 20% of the cells started to exhibit low cell contents. Throughout the course of the experiments the pH in all cultures and at all irradiances was measured and adjusted to between 7.5 and 8.5 (see S1 Fig, supporting information for graphs of pH measurements).

### Direct observations of cell divisions

**Fig 2:**
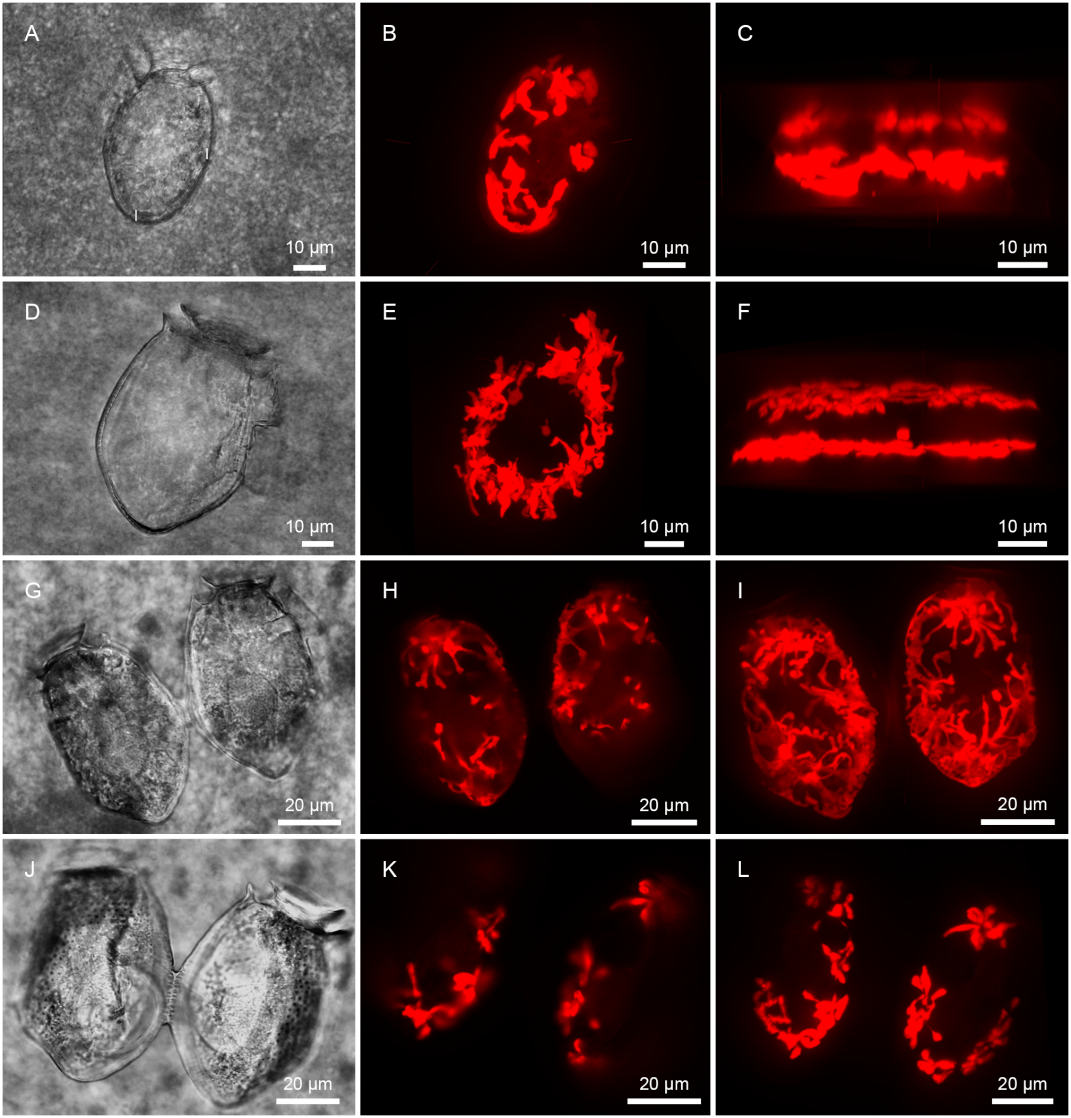
Cell divisions in *D*. *acuminata* (A-C) and *D*. *acuta* (D-L). A, D, G, J light microscopy. H, K, epifluorescense micrographs of kleptochloroplasts. B, C, E, F, I, L, maximum intensity projection of epifluorescence of the kleptochloroplasts. A-C: Same cell, division of *D*. *acuminata* grown at 100_i_, day 3. A-B right lateral view, C dorsal view. D-F: Same cell, division of *D*. *acuta* grown at 100_i_, day 5. D-E right lateral view, F dorsal view. G-I: Same cells, recently divided paired cells of *D*. *acuta* grown at 25_i_, day 12. G-I lateral view. J-L: Same cells, recently divided paired cells of *D*. *acuta* grown at 50_i_, day 63. J-L lateral view.

Despite that more than 680 cells were analyzed with confocal microscopy during the experiments, cells in a clear state of division were only observed nine times (Fig 2). Different stages of cell division were observed; however, the very early stages of cell divisions were difficult to recognize as such. Seven examples of dividing cells were observed and two of these are shown in Fig 2A–2F. Two examples of recently divided paired cells were also observed and examined (Fig 2G–2L). A remarkable observation was the distribution of the kleptochloroplasts in both of the daughter cells during cell division; they were almost complete mirror images of each other. In recently divided cells equal amounts of kleptochloroplast volume, number of kleptochloroplasts and number of kleptochloroplast centers were also observed in the two daughter cells. The measured kleptochloroplast volumes were 4.05 and 3.26 * 10^3^ μm^3^ for the left and right cell in Fig 2I; this difference was not significant when the standard deviation of the sampling is taken into consideration. The cells in Fig 2L also had similar kleptochloroplast volumes, with values of 1.04 and 1.28 * 10^3^ μm^3^ for the left and right cell, respectively. A better visual illustration of these cell divisions and recently divided paired cells is provided as videos of the maximum intensity projection of the epifluorescence of the kleptochloroplasts (3D models) in supporting information (S1–S4 videos). Each of the 3D models is based on z-stacks compiled by 111–164 single micrographs with a distance of 0.2 μm.

### Total volume of kleptochloroplasts

**Fig 3:**
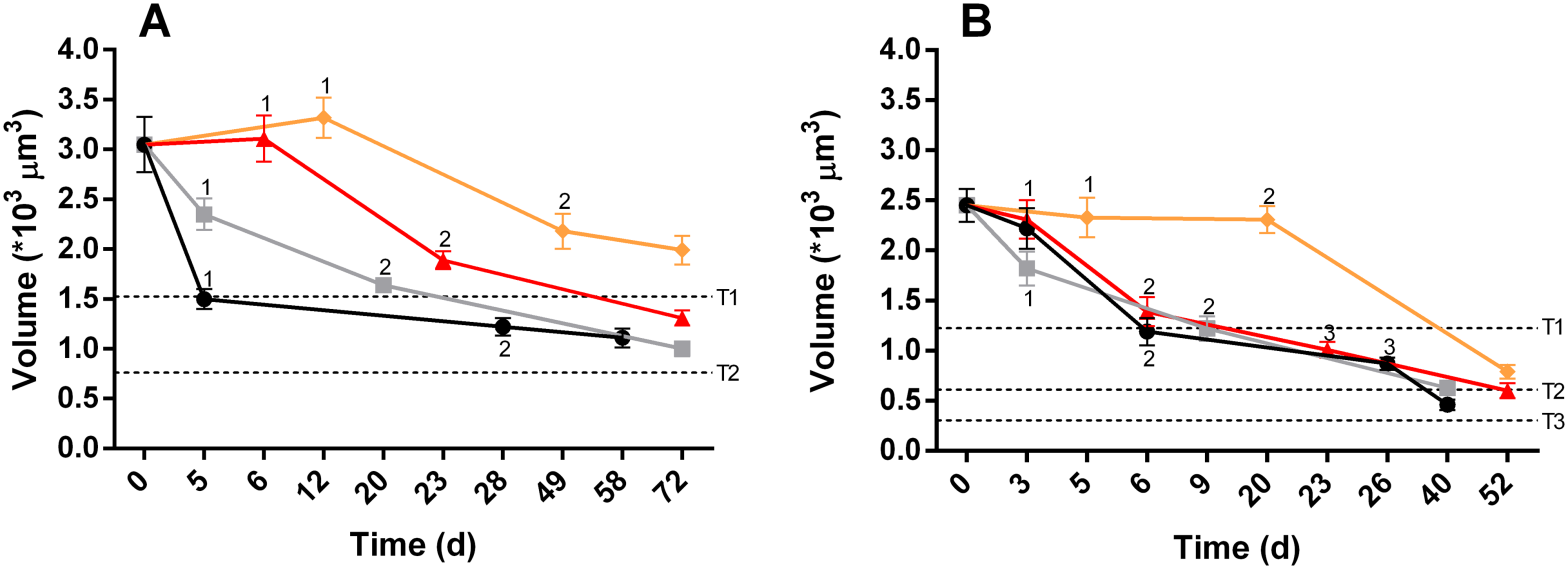
Total kleptochloroplast volume in starved cells of *D*. *acuta* (A) and *D*. *acuminata* (B) at four different light incubations. Each data point represents the mean of 20 cells and the error bars are standard error mean, the different incubations are 100_i_ (black circles), 75_i_ (grey squares), 50_i_ (red triangles), 25_i_ (orange diamonds). Numbers denote the first, second and third cell division. The dashed lines indicate hypothetical trend lines of the expected volumes if *D*. *acuta* and *D*. *acuminata* cannot divide the kleptochloroplast, T1, T2 and T3 is first, second and third division, respectively.

During the experimental period (58–72 days) the total kleptochloroplast volume in *D*. *acuta* decreased in all incubations from an average of 3.05 to 0.80 * 10^3^ μm^3^ per cell (Fig 3A). The hypothetical trend lines (T1 and T2, Fig 3A) show that the decreases are not as low as what would be expected if *D*. *acuta* was not capable of kleptochloroplastidic division. The statistical tests also showed that the total kleptochloroplast volume did not decrease significantly at the first cell division for the two lowest light incubations, 50_i_ and 25_i_ (P>0.05, One-way ANOVA followed by Tukey’s multi comparison). It did decrease significantly at the first cell division for the two other incubations (100_i_ and 75_i_, P≤0.0001 and P≤0.05, respectively). At the second cell division, the total kleptochloroplast volume in the incubations of *D*. *acuta* further decreased though only three of them (75_i_, 50_i_ and 25_i_) had volumes that were significantly lower compared to the previous sampling (P≤0.05, P≤0.001 and P≤0.01, respectively). From the second cell division (day 28–49) until the end of the experiment the total kleptochloroplast volumes at 100_i_ and 25_i_ stagnated, whereas the incubation at 75_i_ decreased significantly (P≤0.05).

The total kleptochloroplast volume in *D*. *acuminata* ranged from an average of 2.45 to 0.46 * 10^3^ μm^3^ per cell during the course of the experiment (Fig 3B). Also for *D*. *acuminata* the hypothetical trend lines (T1, T2 and T3, Fig 3B) show that the decreases are not as low as what would be expected if the cells were not capable of kleptochloroplastidic division. Three of the incubations (100_i_, 75_i_ and 50_i_) showed similar total kleptochloroplast volumes at all the samplings. However, the 100_i_ and 50_i_ incubations had significant decreases in the total kleptochloroplast volume at the second cell division (P≤0.0001 and P≤0.0001, respectively), whereas the 75_i_ incubation decreased the total volume significantly at the first and second division and at the end (P≤0.01, P≤0.05 and P≤0.05, respectively). The largest decline of the total kleptochloroplast volume was observed in the initial phase and after the two first cell divisions, whereafter the volume decreased at a lower rate in the 100_i_, 75_i_ and 50_i_ incubations. Cells in the 25_i_ incubation showed a different tendency and a decrease in total kleptochloroplast volume was not observed at the first and second cell division, though a significant decrease was observed at the end of the experiment (P≤0.0001) (Fig 3B).

### Quantification of kleptochloroplast centers

**Fig 4:**
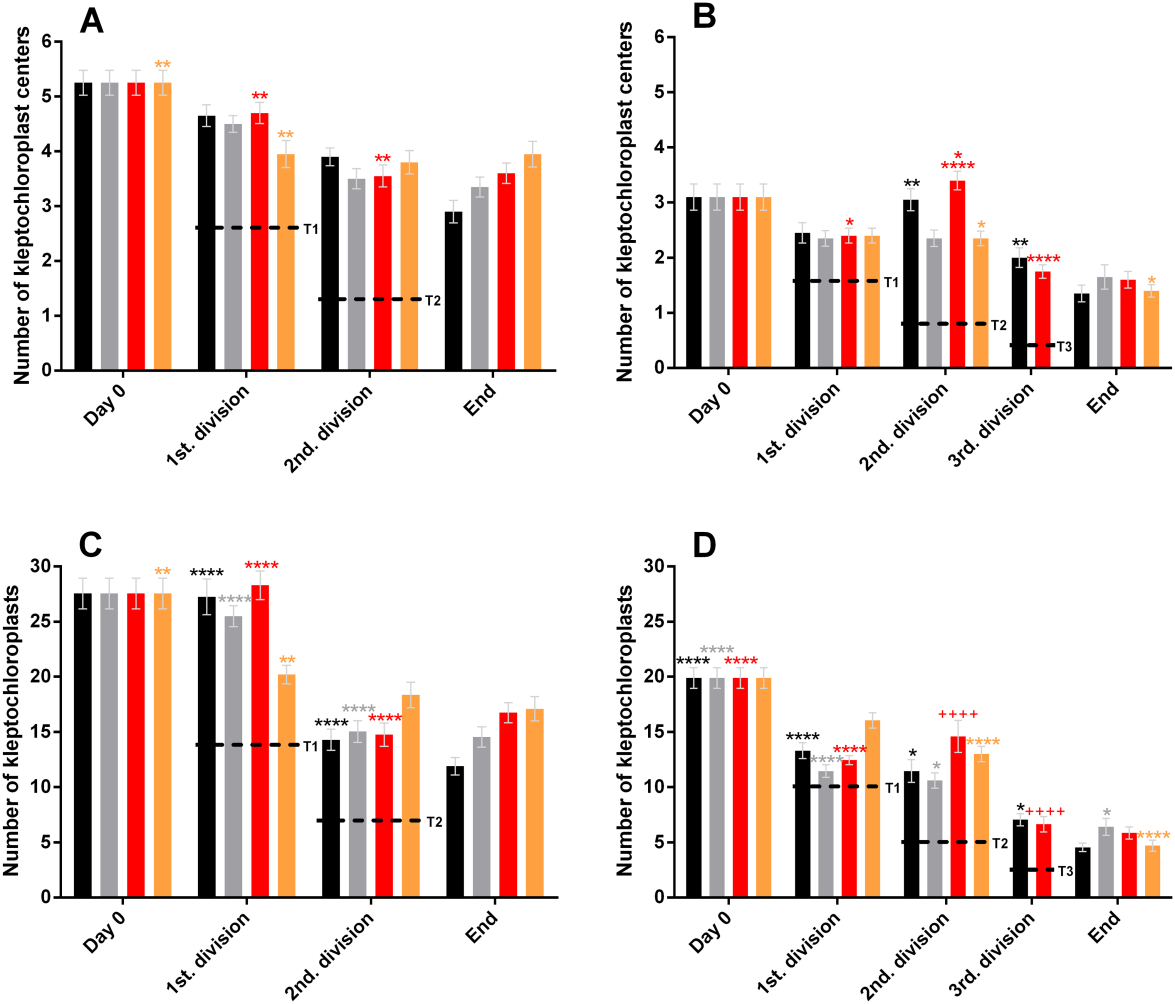
Number of kleptochloroplast centers in *D*. *acuta* (A) and *D*. *acuminata* (B) and total number of kleptochloroplasts in *D*. *acuta* (C) and *D*. *acuminata* (D) during the two experiments at four different light incubations. Each bar represents the mean of 20 cells and the error bars are standard error mean. The incubations are 100_i_ (black bars), 75_i_ (grey bars), 50_i_ (red bars), 25_i_ (orange bars). The asterisks (*) and plusses (+) represent the results of oneway ANOVA, followed by Tukey’s multicomparisons. Identical asterisks or plusses above the bars indicate a significant difference between the samplings, * = (P≤0.05), ** = (P<0.01), *** = (P<0.001), **** = (P<0.0001), ++++ = (P<0.0001). The dashed lines indicate hypothetical trend lines of the expected numbers of kleptochloroplasts and kleptochloroplast centers if *D*. *acuta* and *D*. *acuminata* cannot divide the kleptochloroplast. T1, T2 and T3 indicate first, second and third division, respectively.

In *D*. *acuta* the average number of kleptochloroplast centers decreased from initially 5.3 to 2.9–3.8 per cell at the termination of the experiment (day 58–72), with the highest number observed at the lowest irradiance (Fig 4A). The decreases observed in the number of centers never reached the low levels that were expected (T1 and T2, Fig 4A) if *D*. *acuta* is not capable of dividing the kleptochloroplasts. Only two incubations (50_i_ and 25_i_) decreased the number of kleptochloroplasts centers significantly during the experimental period. In the 50_i_ incubation the significant decrease was seen at the second division (P≤0.001), while the significant decrease in the 25_i_ incubation was already seen at the first division (P≤0.001).

For *D*. *acuminata* the mean number of kleptochloroplast centers decreased from 3.3 to 1.4 per cell in all incubations (day 0–52) (Fig 4B). The decreases observed in the number of centers were not as low as expected (T1, T2 and T3, Fig 4B) if *D*. *acuminata* is not capable of dividing the kleptochloroplasts. At both the first and second cell division no significant decreases were seen in any of the incubations (P>0.05). Instead the 50_i_ incubation increased significantly at the second division (P<0.05). The two incubations that went through a third cell division (100_i_ and 50_i_) experienced a significant reduction in the number of kleptochloroplast centers at that sampling (P≤0.01 and P≤0.0001, respectively). In the 25_i_ incubation a reduction in the number of kleptochloroplast centers (P≤0.05) was observed at the end of the experiment.

### Quantification of kleptochloroplasts

In cells of *D*. *acuta*, the mean total number of kleptochloroplasts was 28 per cell initially, and overall decreases were observed in all incubations (Fig 4C). The decreases were not as low as expected (T1 and T2, Fig 4C) if *D*. *acuta* was not capable of kleptochloroplastidic division, and thereby distributed the number of kleptochloroplasts to the daughter cells upon division. Significant decreases were only observed at one of the divisions for each of the incubations; for 25_i_ the significant decrease (P≤0.01) was observed at the first division (day 12), where it decreased to 20.6 per cell. In the other three incubations (100_i_, 75_i_ and 50_i_) significant decreases were observed at the second division where the number of kleptochloroplasts ranged from 14.7 to 14.2 (P≤0.0001 in all three incubations).

**Fig 5:**
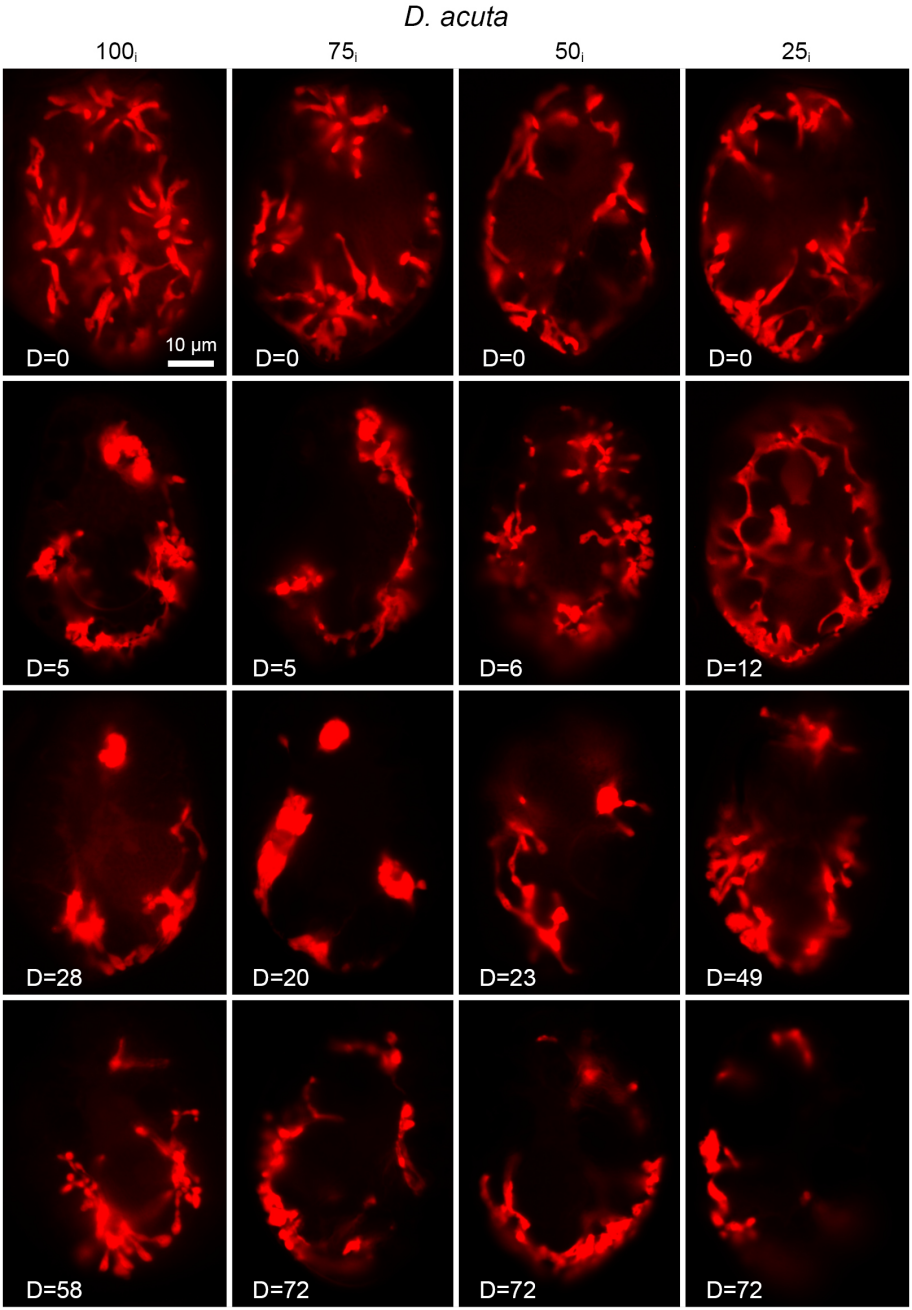
Epifluorescence microscopy of kleptochloroplasts in *D*. *acuta* at the 100_i_, 75_i_, 50_i_ and 25_i_ incubations during the experimental period of 58–72 days. Examples of kleptochloroplasts in one cell are shown from each sampling.

In general, the kleptochloroplasts of *D*. *acuta* became less ramified as the experiment progressed (Fig 5). Fewer ramifications were observed in the 100_i_ and 75_i_ incubations after the first cell division though the number of kleptochloroplasts remained stable. On the other hand, the 50_i_ and 25_i_ incubations maintained high levels of ramifications although the 25_i_ decreased the number of kleptochloroplasts. Further reductions in ramifications and number of kleptochloroplasts were observed after the second cell division in all incubations. Towards the end of the experiment the kleptochloroplasts became visually more intense and concentrated at the root of the centers.

*D*. *acuminata* contained fewer kleptochloroplasts than *D*. *acuta*. The mean number of kleptochloroplasts in *D*. *acuminata* cells decreased from initially 19.9 to 4.6–6.4 per cell at the termination of the experiment (Fig 4D). Also for *D*. *acuminata* the hypothetical trend lines (T1, T2 and T3, Fig 4D) show that the decreases in the number of kleptochloroplasts are not as low as what would be expected if the cells were not capable of kleptochloroplastidic division. At the first cell division, the number of kleptochloroplasts decreased significantly (P≤0.0001) in three of the incubations (100_i_, 75_i_ and 50_i_). At the second cell division, the number of kleptochloroplasts remained constant in all incubations (P>0.05 for all). Two of the incubations (100_i_ and 50_i_) went through a third cell division, where statistically significant decreases were observed for both (P≤0.05 and P≤0.0001). Two incubations of *D*. *acuminata* (75_i_ and 25_i_) experienced a significant decrease in the number of kleptochloroplasts at the termination of the experiment (P≤0.05 and P≤0.0001, respectively).

**Fig 6:**
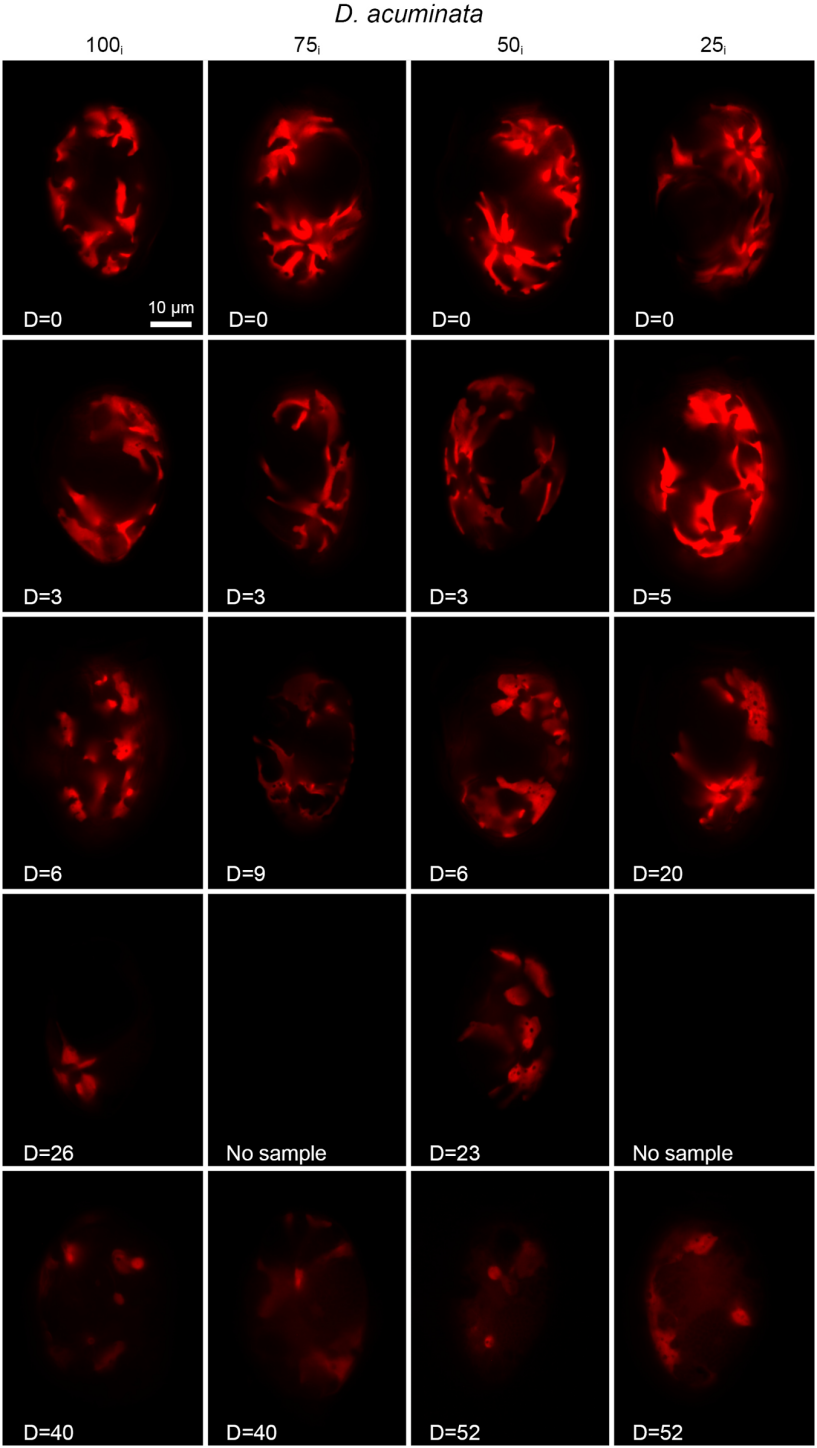
Epifluorescence microscopy of kleptochloroplasts in *D*. *acuminata* at the 100_i_, 75_i_, 50_i_ and 25_i_ incubations during the experimental period of 58–72 days. Examples of kleptochloroplasts in one cell are shown from each sampling.

Reductions in the ramifications of the kleptochloroplasts were observed starting around the second division in all incubations (Fig 6). Cells of the 100_i_ and 75_i_ incubations in general showed fewer ramifications than cells of the 50_i_ and 25_i_ incubations. The kleptochloroplasts, which were initially elongated and thin, became broader and more lobed towards the end of the experiment in both species. In these broader kleptochloroplasts, spherical chloroplast-empty areas could sometimes be observed (e.g. Fig 6, 50_i_, d = 23) and these “dark spots” might indicate storage material (probably lipids and plastoglobulin) within the kleptochloroplasts. These “dark spots” were more abundant in *D*. *acuminata* than in *D*. *acuta*.

**Fig 7:**
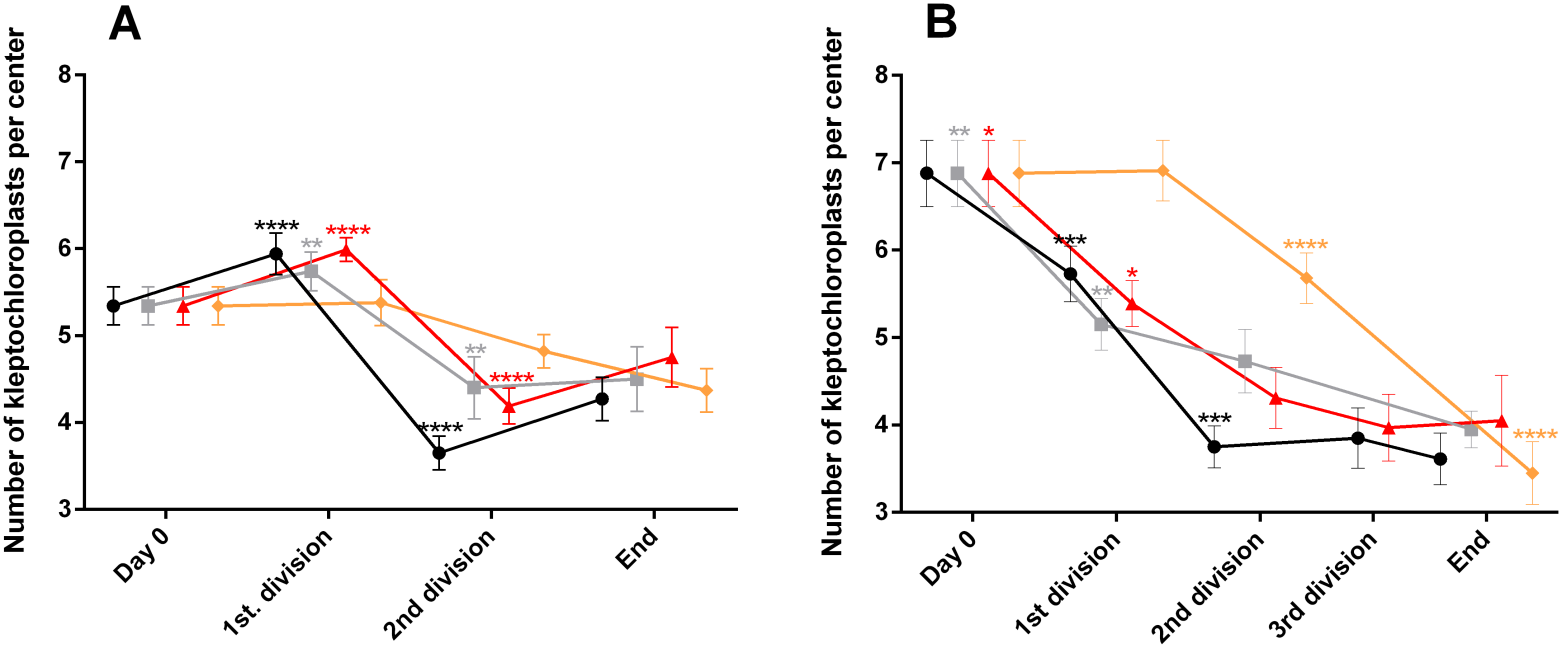
Number of kleptochloroplasts per kleptochloroplast center in *D*. *acuta* (A) and *D*. *acuminata* (B) at four different light incubations. Each data point represents the mean of 20 cells and the error bars are standard error mean. The different incubations are 100_i_ (black circles), 75_i_ (grey squares), 50_i_ (red triangles), 25_i_ (orange diamonds). The asterisks represent the results of the oneway ANOVA, followed by Tukey’s multicomparisons. Identical asterisks above the datapoints indicate a significant difference between the samplings, * = (P≤0.05), ** = (P<0.01), *** = (P<0.001), **** = (P<0.0001).

The number of kleptochloroplasts per center in *D*. *acuta* decreased in all incubations during the experimental period. However, the reduction was moderate and went from initially being on average 5.34 to 4.27–4.75 at the termination of the different incubations (Fig 7A). Significant decreases were observed for three of the incubations (100_i_, 75_i_ and 50_i_) at the second division (P≤0.0001, P≤0.01 and P≤0.0001). No significant difference was found between day 0 and the end in two of the incubations (75_i_ and 50_i_).

The number of kleptochloroplasts per center in *D*. *acuminata* cells was higher than in *D*. *acuta* at the start of the experiments (6.88 vs. 5.34, Fig 7B). The mean number of kleptochloroplasts per center in *D*. *acuminata* decreased from initially being 6.88 to 3.45–4.05 per cell at the end of the experiment (Fig 7B). After the first cell division, a significant decrease in the number of kleptochloroplasts per center in *D*. *acuminata* was observed in two of the incubations (75_i_ and 50_i_; P≤0.01 and P≤0.05, respectively), whereas this reduction was not observed until after the second cell division in cells at 100_i_ (P≤0.001). A significant reduction in the number of kleptochloroplasts per center in the 25_i_ incubation was not observed until after the second cell division (P≤0.0001).

## Discussion

### Division of kleptochloroplasts in *Dinophysis*

This study documented for the first time division of the kleptochloroplasts and kleptochloroplast centers in *D*. *acuta* and *D*. *acuminata*. Both quantitative results and the photo documentations clearly demonstrated that the reductions in the number of kleptochloroplasts and kleptochloroplast centers during long term incubations were not caused by cell division. This supports our H1 hypothesis and contradicts earlier studies by Minnhagen et al. [5–6].

In all the incubations reductions of the number of kleptochloroplasts and kleptochloroplast centers as well as total kleptochloroplast volume were observed, although these could not be linked to cell division. These reductions were more pronounced in the high irradiance incubations (100_i_ and 75_i_) as compared to at the low irradiance incubations. Both H2 and H3 can therefore be accepted. The results of this study are therefore in accordance with a recent study on photoregulation in prey starved cells of *D*. *acuta* [12]. These results imply that we need to look at *Dinophysis* spp. and other similar kleptochloroplastidic species in a new perspective and explore how this extensive control is possible in order to understand the complex interactions, implications and evolutionary relationships.

### Degradation of kleptochloroplasts in *Dinophysis*

In both *Dinophysis* species in the present study the kleptochloroplast volumes, number of kleptochloroplasts and number of kleptochloroplast centers in all incubations decreased to the same minimum level towards the end of the experiments. The incubations differed mainly in that cells grown at the highest light intensities reached this threshold faster than cells grown at lower irradiance. These findings indicate that a degradation of the kleptochloroplasts takes place and that this degradation could correlate with time of starvation in combination with irradiance instead of cell divisions. The apparent degradation of the kleptochloroplasts during starvation could be caused by some external constraints, such as resource limitations, e. g. nitrogen and phosphorous.

We used a rich algal growth medium (f/2), which contains high levels of major nutrients (nitrate and phosphate), micronutrients and vitamins, so *Dinophysis* was principally not nutrient limited. However, recent studies have shown that *Dinophysis acuminata* does not take up nitrate [14,15]. It is well-known that nitrogen availability for example determines the cellular content of light-harvesting chlorophyll protein complexes [16]. This raises the question of whether the *Dinophysis* cultures became nitrogen limited when starved of prey in our experiments. Hattenrath-Lehmann & Gobler (2015) found that cultures of *D*. *acuminata* grew better both with and without prey when ammonium, glutamine and organic matter from a sewage effluent were added to the cultures and this suggested direct nitrogen assimilation [15]. However, *D*. *acuminata* cultures could not grow for an extended period of time without prey even if supplied with ammonium or organic nitrogen forms [15]. In the present study and in the study by Hansen et al. [12], lipids and starch were observed to build up in cells, indicating accumulation of organic carbon. Further studies on the storage products, nutrient assimilation and gene expression during prey starvation are required to fully understand the possible need to acquire nutrients from the prey.

### Kleptochloroplastidic control

How species of *Dinophysis* are capable of controlling kleptochloroplastidic division is yet unknown, but at least two explanations are possible: (1) *Dinophysis* spp. has kept chloroplast regulating genes from earlier steps in the evolution when ancestors were photoautotrophic and/or, (2) regulating genes have been incorporated into their genome by horizontal gene transfer (HGT) from the prey. Most of the research on chloroplast origin in dinoflagellates has been on autotrophic species, but it is believed that non-photosynthetic dinoflagellates have lost the original chloroplasts at some point in evolution [1]. If this is the case, genetic traces of plastids could potentially be left in the genome of *Dinophysis*. This might be the case for the heterotrophic dinoflagellates *Crypthecodinium cohnii* and *Oxyrrhis marina*. Several plastid-linked protein coding sequences have been found in both species. In *C*. *cohnii* ten plastid targeted genes were found [17], and eight in *O*. *marina* [18]. In similar studies on the genome of *D*. *acuminata* only five nuclear-encoded, chloroplast-targeted genes were found. The genes found had multiple algal origins (peridinin and fucoxanthin dinoflagellates and haptophyte), but one of them was identical to those present in cryptophytes [4].

The multiple algal sources of chloroplast encoded genes that were found in *D*. *acuminata* are thought to indicate the occurrence of HGT rather than being evolutionary remnants. HGT of plastid related genes has also been shown to be widespread within the Chromalveolates [19]. It is therefore possible that transfer of chloroplast encoded genes from prey organelles could have occurred in *Dinophysis* spp., although the actual amount of chloroplast encoded genes is minimal when compared to the chloroplast related genes of fully autotrophic algae [4]. Further research on chloroplast encoded genes of *Dinophysis* spp. is therefore needed to explain the kleptochloroplastidic control. It might be advantageous to search for the transcribed genes in starved cells compared to well fed.

## Perspective

Protists that are capable of utilizing the kleptochloroplasts from their prey are quite common in marine waters. These organisms also sequester other cell organelles as well, including prey nuclei allowing them to exploit their algal prey for a considerable amount of time; e.g. *Mesodinium rubrum* [20]. *Dinophysis*, and the closely related genus *Phalacroma*, are unique among the dinoflagellates in that they exclusively retain the chloroplasts [21–23]. The oligotrich ciliates represent another group of protists that contains several genera that sequester kleptochloroplasts exclusively, e.g. *Cyrtostrombidium* spp, *Laboea strobila*, some *Strombidium* spp., and *Tontonia* spp. [2]. While *Dinophysis* spp. are quite restricted in the type of chloroplasts they can exploit, the oligotrich ciliates seem to be able to acquire kleptochloroplasts from a variety of algal groups. The drawback of exclusive retention of chloroplasts is that control of the chloroplast is limited and retention of functional chloroplasts is relatively short [24]. The capability of dividing the kleptochloroplasts without any nuclear material from a prey, such as observed in the genus *Dinophysis*, has not previously been documented in any other kleptochloroplastidic protist. Thus, *Dinophysis* spp. may serve as a model organism in studies of chloroplast acquisition and the molecular mechanisms involved.

## Supporting information

S1 Fig pH values of *D*. *acuta* (A) and *D*. *acuminata* (B) at four different light incubations during the course of the experiments. The different incubations are 100_i_ (black circles), 75_i_ (grey squares), 50_i_ (red triangles), 25_i_ (orange diamonds).
(DOCX)

S1 video Maximum intensity projection of the epifluorescence of the kleptochloroplasts in a cell division of *D*. *acuminata* grown at 100i, day 3. (MPG)

S2 video Maximum intensity projection of the epifluorescence of the kleptochloroplasts in a cell division of *D*. *acuta* grown at 100i, day 5. (MPG)

S3 video Maximum intensity projection of the epifluorescence of the kleptochloroplasts in recently divided paired cells of *D*. *acuta* grown at 25i, day 12. (MPG)

S4 video Maximum intensity projection of the epifluorescence of the kleptochloroplasts in recently divided paired cells of *D*. *acuta* grown at 50i, day 63. (MPG)
