# Supplementary material for: Evolutionary transition towards permanent chloroplasts? - Division of kleptochloroplasts in starved cells of two species of Dinophysis (Dinophyceae)
Source: PLoS One. 2017 May 11;12(5):e0177512. doi: 10.1371/journal.pone.0177512 (PMC5426790; doi:10.1371/journal.pone.0177512)
Supplement: S1 Fig — The different incubations are 100i (black circles), 75i (grey squares), 50i (red triangles), 25i (orange diamonds). (DOCX) [file pone.0177512.s001.docx]

**S1 Fig**





S1 Fig: pH values of *D. acuta* (A) and *D. acuminata* (B) at four different light incubations during the course of the experiments. The different incubations are 100_i_ (black circles), 75_i_ (grey squares), 50_i_ (red triangles), 25_i_ (orange diamonds).
